# Occurrence of Mental Illness and Mental Health Risks among the Self-Employed: A Systematic Review

**DOI:** 10.3390/ijerph18168617

**Published:** 2021-08-15

**Authors:** Kristina Willeke, Patrick Janson, Katharina Zink, Carolin Stupp, Sarah Kittel-Schneider, Anne Berghöfer, Thomas Ewert, Ryan King, Peter U. Heuschmann, Andreas Zapf, Manfred Wildner, Thomas Keil

**Affiliations:** 1 State Institute of Health, Bavarian Health and Food Safety Authority, 91058 Erlangen, Germany; patrick.janson@lgl.bayern.de (P.J.); katharina.zink@lgl.bayern.de (K.Z.); Carolin.Stupp@lgl.bayern.de (C.S.); thomas.ewert@lgl.bayern.de (T.E.); manfred.wildner@lgl.bayern.de (M.W.); thomas.keil@lgl.bayern.de (T.K.); 2 Institute of Clinical Epidemiology and Biometry, University of Würzburg, 97070 Würzburg, Germany; e_heuschma_p@ukw.de; 3 Department of Psychiatry, Psychotherapy and Psychosomatic Medicine, University Hospital Würzburg, 97080 Würzburg, Germany; Kittel_S@ukw.de; 4 Institute of Social Medicine, Epidemiology and Health Economics, Charité—Universitätsmedizin Berlin, 10117 Berlin, Germany; anne.berghoefer@charite.de (A.B.); Ryan.king@charite.de (R.K.); 5 Clinical Trial Center Würzburg, University Hospital Würzburg, 97080 Würzburg, Germany; 6 Bavarian State Ministry of the Environment and Consumer Protection, 81925 Munich, Germany; Andreas.Zapf@stmuv.bayern.de; 7 Pettenkofer School of Public Health, University of Munich, 81377 Munich, Germany

**Keywords:** incidence, mental disorders, mental health, mental illness, prevalence, self-employed, small business, systematic review

## Abstract

We aimed to systematically identify and evaluate all studies of good quality that compared the occurrence of mental disorders in the self-employed versus employees. Adhering to the Cochrane guidelines, we conducted a systematic review and searched three major medical databases (MEDLINE, Web of Science, Embase), complemented by hand search. We included 26 (three longitudinal and 23 cross-sectional) population-based studies of good quality (using a validated quality assessment tool), with data from 3,128,877 participants in total. The longest of these studies, a Swedish national register evaluation with 25 years follow-up, showed a higher incidence of mental illness among the self-employed compared to white-collar workers, but a lower incidence compared to blue-collar workers. In the second longitudinal study from Sweden the self-employed had a lower incidence of mental illness compared to both blue- and white-collar workers over 15 years, whereas the third longitudinal study (South Korea) did not find a difference regarding the incidence of depressive symptoms over 6 years. Results from the cross-sectional studies showed associations between self-employment and poor general mental health and stress, but were inconsistent regarding other mental outcomes. Most studies from South Korea found a higher prevalence of mental disorders among the self-employed compared to employees, whereas the results of cross-sectional studies from outside Asia were less consistent. In conclusion, we found evidence from population-based studies for a link between self-employment and increased risk of mental illness. Further longitudinal studies are needed examining the potential risk for the development of mental disorders in specific subtypes of the self-employed.

## 1. Introduction

A considerable proportion of the global workforce consists of self-employed individuals [1]. In 2018, the proportion of self-employed among the Organisation for Economic Cooperation and Development (OECD) countries varied from 6% in the United States, 32% in Spain and to 52% in Columbia [2]. Some individuals actively choose self-employment for the greater independence compared to being an employee, whereas for others self-employment is the only way out of unemployment [3]. However, both groups are exposed to economic uncertainties [3,4]. Due to their autonomy, the self-employed may be more committed and have a higher job satisfaction in comparison to employees, but often they are also exposed to more work–family conflict and longer working hours [5]. The more unregulated the market, the higher the competitive pressure tends to be for the self-employed. Sickness presenteeism is a considerable health risk and seems to be displayed more often by the self-employed [6]. These conditions may cause chronic stress leading to negative mental and/or physical health effects [3].

Mental illness, with a 12-month prevalence of 18–30% globally and 27% in Europe is the primary reason for prolonged sick leave in the OECD-countries [6,7]. Thus, a large proportion of costs are explained by absenteeism or a loss of productivity due to mental disorders [7]. According to Leignel et al., 80% of all American employed and self-employed workers experienced work-related stress [8]. Apart from working hours, general job and income uncertainties, psychosocial stress may also be related to high job requirements, insufficient human resources and insufficient training and skills to meet work-related challenges [8]. Perceived psychosocial stress can lead to mental health problems, increased absenteeism and decreased productivity [8,9,10]. Some studies showed that the self-employed, especially small business owners, may experience more negative stress than employed workers [11,12,13,14]. Besides other mental health problems [11], a possible consequence of stress at the job is depression, a condition that has been increasing in recent decades [7,15]. Whether the prevalence of anxiety and depressive disorders is higher or lower among the self-employed compared to employed workers seems unclear [15,16,17,18].

Suicide is significantly associated with mental disorders, with over 90% of affected individuals having suffered from a mental disorder, such as affective disorders, schizophrenia or personality disorders [19,20,21]. Occupational risk factors for suicide include high work demands or overwork in combination with a low level of job autonomy, or financial problems [19,22]. There are some indications for more suicidal ideation or suicides among the self-employed [23,24].

In general, the current literature about the extent of mental health problems among the self-employed is heterogeneous, incomplete and partly contradictory [3,25]. A stringent systematic review including adequate population-based studies as a basis for the development of targeted prevention strategies is lacking. Provisions in occupational health and safety legislation, such as regular risk assessments and occupational health management, generally do not cover the self-employed. Self-employment and atypical employment are increasing worldwide at the same time as rapid technological and social change and increasing global competition, thus requiring special attention from social policy and preventive medicine decision makers [26].

Therefore, the purpose of the present systematic review was to identify all studies worldwide that examined the occurrence of mental health problems amongst the self-employed sole proprietors and small business owners, evaluate their quality and summarize the findings for each specific mental disease.

## 2. Materials and Methods

For the present systematic review, we adhered to the guidelines of the Cochrane Handbook for Systematic Reviews of Interventions version 6.0 [27] and the recommendations of the Preferred Reporting Item for Systematic Review and Meta-Analysis (PRISMA) statement [28]. Since our study did not involve human contact and was based on previously published studies, ethical approval was not required.

### 2.1. Search Strategy

We searched the PubMed, Web of Science and Embase databases up to March 2020 without restrictions regarding languages or year of publication. For PubMed the following search string was used: “((“Small Business” [Mesh]) OR “self-employed”) Sort by: Best Match Filters: Humans “. For the Web of Science database, we used the following search string: “TS = “Small business *“ OR TS = “small enterprise *“ OR TS= “micro business *“ OR TS = “small and medium-sized enterprise*“ OR TS = “self-employed“ OR TS = “freelanc *“ OR TS = “independent *“ OR TS = “free agent *“ AND TS = Disease * OR TS = illness * OR TS = sickness * OR TS = “ill health“ OR TS = ailment * OR TS = malad * OR TS = disorder * OR TS = complaint * OR TS = “health problem*“ OR TS = strain OR TS = “health burden“ OR TS = Health * OR TS = “well-being“ OR TS = “physical constitution *“ OR TS = “psychological constitution *“ OR TS = “state of health“ OR TS = Presenteeism OR TS = absenteeism OR TS = truancy”. For the Embase database, we used the following search string: “(’small business *’ OR ’small enterprise *’ OR ‘micro business *’ OR ’small and medium-sized enterprise *’ OR ’self employed *’ OR ‘freelanc *’ OR ‘free agent *’) AND (disease * OR illness * OR sickness * OR ‘ill health’ OR ailment * OR malad * OR disorder * OR complaint * OR ‘health problem*’ OR strain * OR ‘health burden *’ OR health * OR ‘well being *’ OR ‘physical constitution *’ OR ‘psychological constitution *’ OR ’state of health’ OR presenteeism OR absenteeism OR ‘truancy’) AND [embase]/lim”.

On 7 April 2021, we conducted an update of our systematic literature search in PubMed for the last 13 months using the previous search string, but adding a filter focusing only on cohort studies.

Supplementary to the systematic search in major databases, we further identified additional studies by screening the references of all selected articles and online abstracts of conferences.

### 2.2. Inclusion and Exclusion Criteria

There is no universal definition of self-employment or small enterprises. Therefore, to avoid excluding potential studies, we used a broad definition of “self-employment” including everyone who works on his/her own in a sole proprietorship, or who is an entrepreneur of a small business with or without the employment of other persons. A priori, we did not define the size of a small business, but followed the definitions of the authors of the studies. The self-employed had to run their own business as their main jobs.

We included primary observational studies of high quality, such as systematic reviews with or without meta-analysis, as well as original articles, cross-sectional and cohort studies that published quantitative results. Articles were considered as relevant if self-employment was examined or mentioned as an influencing factor in a mental health outcome. The mental health outcomes included depression or depressive disorders, anxiety or neurotic symptoms, suicide or suicide attempt, and further mental disorders such as psychosis/schizophrenia, attention deficit hyperactivity disorder (ADHD), bipolar affective disorder, posttraumatic stress disorder, substance use disorder, personality disorder, somatoform disorder, persistent affective disorder and depersonalization. We also included aspects of the ICD-10 Z codes which indicate risk factors or factors influencing contact with health services such as stress or distress, perceived mental health, burn-out-syndrome/exhaustion, poor mental well-being, feeling sad/worried.

The excluded study designs were intervention studies, qualitative studies, narrative reviews, case reports and series, book chapters, editorials and letters. Furthermore, a study was excluded if it investigated a convenience sample, employees only, or physical conditions only. We also excluded “risky health behavior” such as smoking, alcohol or drug consumption, unhealthy eating habits, obesity and poor life satisfaction, because we wanted to focus on mental illnesses and disorders rather than unhealthy lifestyle behavior. We also excluded studies that did not include employed workers as a reference group or that were rated as low or moderate quality studies.

### 2.3. Assessment of Studies

The screening of titles and abstracts of the identified articles was performed by the first author (KW) taking into account the inclusion and exclusion criteria defined above. Duplicates of studies and studies that were not available via the German library network ’subito’ were not considered.

For full text screening, non-English and non-German publications were translated by external native-level speaking scientists or professional translators into English or German. The screening of the full texts was conducted independently by two of the authors, and in case of discrepancies a third author was consulted. Subsequently, studies were assessed for risk of bias (see quality assessment below).

The data extraction of all included studies was conducted by the first author (KW) and subsequently double-checked by a second author (PJ, KZ). The very few disagreements were solved by consulting a third author (TK). From the included studies, we extracted the following parameters: first author, publication data, country, study design, sample size, response rate, percentage of female participants, age of the study population, origin and main characteristics of the target group, occupational status, assessment tools and main results. In case of missing data or other queries, we did not contact the authors of the included publications.

### 2.4. Quality Assessment

The quality of the studies was rated based on the slightly modified assessment tool that was developed by Hoy et al. and showed a high interrater agreement [29]. It contains nine items (Supplementary Table S1) of potential study bias plus a summary assessment regarding the internal and external validity of the study. The assessment of the external validity included three items: representation, sampling and random selection. The internal validity was determined using the following six items: nonresponse bias, data collection, case definition, reliability/validity of tool, method of data collection, numerator(s) and denominator(s).

The overall quality was scored from 0 (no risk of bias) to 9 (highest risk of bias) and described by the following three categories: low risk (0–2 points); moderate risk (3–5) and high risk (6–9).

**Figure 1:**
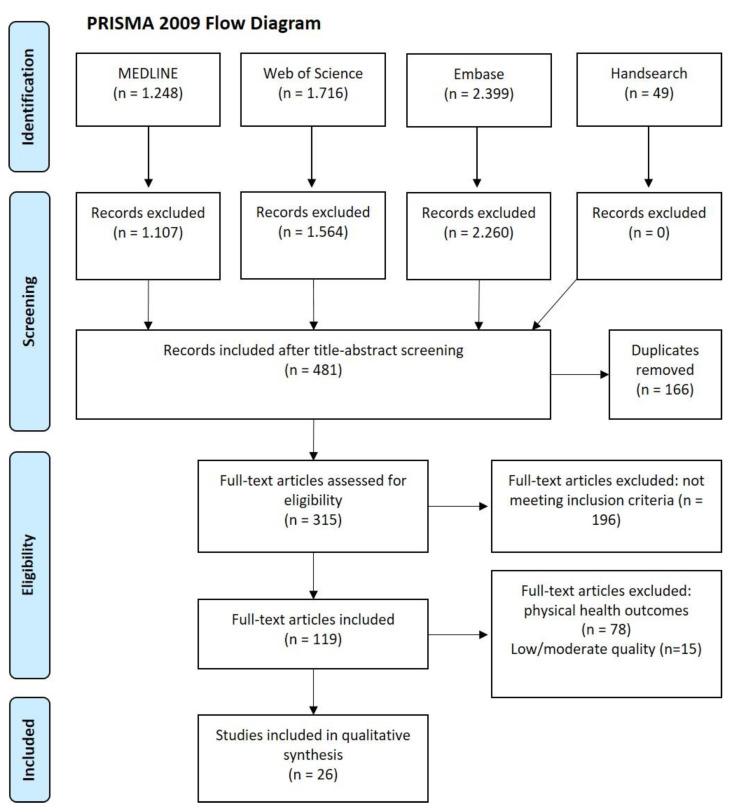
Prisma Flow Chart showing the selection process of studies on mental health in the self-employed versus employees [28].

For studies without prevalence estimates, we did not consider the last item (“Were the numerator(s) and denominator(s) for the parameter of interest appropriate?”). In this case, the classification of the study quality was described as follows: low risk (0–2), moderate risk (3–5) and high risk (6–8) (Table S1). For the present review, two of the authors independently rated each study as either low, moderate or high risk of bias. The overall interrater agreement with this assessment tool for the study quality was 91%. The very few disagreements were solved by consulting a third author. Based on the overall quality scores we included only longitudinal and cross-sectional population-based studies with a low risk of bias in the present systematic review (Figure 1).

## 3. Results

### 3.1. General Study Characteristics

We identified 5412 potential publications for our research question. After removing duplicates, screening the full texts and applying our inclusion and exclusion criteria we were able to include 26 primary studies (three longitudinal, 23 cross-sectional), but no systematic review (Figure 1). Among the excluded articles were 15 studies that we did not consider due to concern of potential bias (Figure 1 step eligibility; Tables S2–S4). Quality assessment of the included studies with good quality is shown in Table 1 in detail.

In total, the 26 studies collected data from 3,128,877 participants. The identified studies were conducted in 15 countries covering all continents except Africa. They were predominantly from Europe and Asia, and most of them were large-scale investigations including register data analyses and national surveys. All included studies were published between 2000 and 2019 (Table 2 and Table 3).

### 3.2. Study Population Characteristics

Overall, the studies investigated a similar proportion of men and women; covering an age range from 15 to 65 years. The two largest studies were based on analyses from national register data in Sweden, and from a large German insurance company, with over one million study participants each. The various survey populations included up to 68,000 participants. Only three studies examined less than 1000 subjects (Table 2 and Table 3). Some studies adjusted their results for potential confounders such as age and socioeconomic status, whereas others did not.

### 3.3. Assessment of Employment Categories

The included studies used self-employment definitions such as sole proprietors (in general or by a specific profession), and business owners (i.e., employers with own employees), and without any specification. Common categories of employed persons included white- and blue-collar workers (referring to non-manual and manual workers respectively), but also skilled/unskilled, and with/without responsibilities. Swedish register studies differentiated high-level non-manual (with ≥3 years of post-secondary school education) from low-level non-manual (<3 years of post-secondary school education) employees and high-level manual (with ≥2 years of post-secondary school education) from low-level manual (<2 years of post-secondary school education) workers. Very few studies investigated only one occupational group, such as physicians [33,36], whereas most of the included studies combined and evaluated all professions together (Table 2 and Table 3).

The employment status (including self-employment) was assessed by self-report or (mostly in the larger studies) by information from register and insurance data.

The self-employed groups were defined in various ways across the studies: (i) all self-employed, (ii) only sole proprietors (i.e. without personnel), (iii) small business owners (e.g. <10 employees), (iv) specific occupational groups such as medical doctors or farmers, and (v) not precisely defined (Table 2 and Table 3).

Across the studies, various employment definitions were used as reference categories for the comparisons of mental health outcomes with the self-employed. The reference categories of employees in the three longitudinal studies were: (i) middle level non-manual (“white-collar”) employees, (ii) high level non-manual (“white-collar”) employees (but incidence rates were compared with four different categories of manual and non-manual employees) and (iii) all full-time permanent employees (Table 2).

### 3.4. Outcome Measures

The included studies investigated various mental health outcomes including mental illness (at least one diagnosis) (three studies), depression and suicidal thoughts (seven and two studies resp.), anxiety (two studies), stress (6 studies), burnout and exhaustion (one each) as well as self-rated poor general mental health outcomes (eight studies). They have been defined in various ways using different data sources and assessment tools (Table 2, Table 3 and Table 4).

### 3.5. Incidence of Mental Disorders in Longitudinal Studies

We identified only three longitudinal studies of good quality (two from Sweden, one from South Korea).

The largest and longest study with over one million subjects and 25 years of follow-up was a nation-wide Swedish register and census-based study. In this study, Tiikkaja et al. showed that the proportion of the self-employed who were admitted to hospitals for mental disorders was higher than the proportion of white-collar employees. However, when compared with blue-collar employees the self-employed had a considerably lower incidence of hospital admissions for mental disorders [30].

The second largest study, a 15-year follow-up analysis of the national Swedish twin registry, found a lower incidence of disability pensions due to mental diagnoses in the self-employed compared to white- and blue-collar employees [16].

The smallest and relatively shorter longitudinal analysis was based on a prospective cohort study from South Korea with a 6-year follow-up. Stratified for men and women, Jang et al. reported similar adjusted effect estimates of severe depressive symptoms in the self-employed compared to white- and blue-collar workers [31] (Table 2 and Table 4).

### 3.6. Prevalence of Mental Disorders in Cross-Sectional Studies

Most of the cross-sectional studies were from Europe and Asia, only three from the U.S.A. and one from Australia. The European cross-sectional studies of good quality that were published within the last 10 years suggested a lower prevalence of depression and anxiety among the self-employed vs. employees, whereas those that were published before 2010 showed more negative mental health effects for the self-employed. Almost all Asian and North American cross-sectional studies reported a higher prevalence in the self-employed with varying mental disorders, regardless of the decade when they were published (Table 2 and Table 4).

#### 3.6.1. Mental Illness (at Least One)

One cross-sectional study that investigated a variety of aggregated mental disorders showed lower risks for mental and especially somatoform disorders for the self-employed versus employed persons, with similar risks for affective disorders and substance abuse [25] (Table 4 and Table 5).

#### 3.6.2. Depression

Out of five studies assessing depression or depressive symptoms, one study from Asia described a higher prevalence among the self-employed and small business owners (15.9%) compared to full-time employees (12.1%) [17]. In Europe, Stahmeyer et al. showed similar findings for both male and female self-employed in 2006, whereas in a more recent investigation in 2015 the same authors found a lower prevalence in both sexes (Table 4 and Table 6) [15]. Another study from Europe, by Combois et al., found rather contradictory results regarding sex. Self-employed men were less affected by depression than employed men whereas the opposite effect was seen in self-employed vs. employed women [32] (Table 4 and Table 6).

#### 3.6.3. Suicidal Thoughts

We did not find any study that examined the prevalence of suicide or attempted suicide in the self-employed compared to employed persons. However, two surveys from South Korea examined suicidal thoughts (ideation). One of them showed a significantly higher prevalence of suicidal thoughts among the self-employed compared to employees for both men (5.4% vs. 3.7%) and women (11.3% vs. 8.3%) [24]. The other one, on the contrary, showed a lower prevalence among the self-employed versus paid workers for both men and women [40]. The latter specifically examined sales and service workers as subgroups of self-employed and employees (Table 4 and Table 7).

#### 3.6.4. Anxiety

The two cross-sectional studies that examined anxiety used different assessment tools (Table 3). They found no considerable differences in anxiety levels among the self-employed in comparison to their employed reference groups [25,46] (Table 4 and Table 8).

#### 3.6.5. Stress, Exhaustion, Burnout

Three out of six studies that compared stress levels of the self-employed and employees, showed higher psychosocial stress levels among the self-employed in comparison to different reference groups of employed persons [11,13,14]. Two studies that investigated stress among the self-employed used questionnaires specially developed for the study that were not validated [13,14].

Stress levels were slightly higher among the self-employed without employees in comparison to those with employees, but both self-employed groups were more affected than the employed [14]. By contrast, sole proprietors (i.e., self-employed without employees) in an Australian study showed lower stress levels compared to employees [45]. One study reported lower values of stress among self-employed in comparison to employees [33] (Table 4 and Table 9).

Only one study (from Europe) investigated burnout. It reported a lower occurrence of burnout among physicians who were self-employed compared to those who were employed [36] (Table 4 and Table 9).

In a Swedish study, less exhaustion was found among self-employed men compared to permanent and temporary employed men, whereas among women there was no considerable difference [35] (Table 4 and Table 9).

#### 3.6.6. Self-Rated Poor General Mental Health

Two of the eight cross-sectional studies that examined self-rated poor general mental health found no difference [43,44], whereas four found worse [18,38,39,41,43] and one better general mental health outcomes among the self-employed compared to employees [34]. A survey from Finland differentiated between the two main types of self-employment. The authors found that sole proprietors had a higher prevalence of self-reported depression or anxiety, whereas the self-employed with employees had a lower prevalence compared to employees [37]. Five studies used similar validated questionnaires or interviews asking for general health with a response rate ranging from 42 to 80% (Table 4 and Table 10).

## 4. Discussion

### 4.1. Main Findings

After a comprehensive screening and a stringent evaluation process, we identified 26 population-based studies of good quality from across the globe assessing and comparing incidence and prevalence of mental illnesses between the self-employed and employees. Only three of these studies were longitudinal studies.

Evidence from the longitudinal studies in Europe showed that the self-employed had a lower incidence of mental disorders compared to blue-collar workers, whereas compared to white-collar workers the results were inconsistent. The Asian longitudinal study showed no difference in mental disorders between the self-employed and employees. However, it did not differentiate between blue- and white-collar workers.

Most of the Asian cross-sectional studies showed a higher prevalence of mental disorders among the self-employed compared to employees. This was also true for the older European cross-sectional studies. However, most of the European cross-sectional studies from the last decade found lower or similar prevalence estimates among the self-employed. The majority of the American and Australian cross-sectional studies found a similar prevalence of mental disorders among the self-employed and the employees.

### 4.2. Comparison of Studies

#### 4.2.1. Longitudinal Studies

The results of the three longitudinal studies included in this review were partly inconsistent. Whereas both Swedish longitudinal register studies showed that the self-employed were less likely to develop mental disorders than blue-collar workers their results regarding the comparison of the self-employed’s mental health with white-collar employees seemed contradictory. Different definitions of the outcome in the two studies may have been responsible. The national registry study by Tiikkaja et al., which analyzed data from the entire Swedish population, showed that self-employed had a higher incidence of hospitalization for a psychiatric diagnosis than white-collar employees [30]. In contrast, the twin registry study by Samuelson et al. showed that self-employed workers were less likely to receive a disability pension due to psychiatric illness than white-collar employees [16]. One possible explanation for the difference could be that the self-employed with mental disorders are less likely to file disability pension claims than employees. Employees with mental illnesses may receive assistance in filing applications through intervention from their company by supervisors, colleagues or company medical officers. This may lead to more frequent or earlier recognition compared to the self-employed who lack this workplace environment for support. The amount of the expected disability pension may also play a role. In Sweden, the self-employed appear to have twice the poverty rate (defined as below 60% of average income) than employees [47]. Since a disability pension represents only about 80% of the average income [48], the associated financial losses could be another explanation for why the self-employed are less likely to file such claims than employees.

The comparison of the two Swedish studies showed an overall better methodological quality of the national register study by Tiikkaja et al. (Table 1). The authors of the twin registry evaluation, Samuelson et al., pointed out several fundamental methodological limitations of their study. In particular, the results for mental diagnoses should be interpreted cautiously because the precision of the outcome estimators was low (as indicated by relatively wide confidence intervals). They also emphasized the regional differences in Sweden with respect to disability pensions. For example, the incidence for disability pensions due to mental health diagnoses was higher in western and southern Sweden than in the rest of the country. Despite adjustment for age, sex and family factors, the reasons for this difference remain unclear. There is evidence that the type of treatment, forms of rehabilitation, or decisions of local social insurance agencies about the granting of disability pension may differ between regions [49,50]. In addition, it should be emphasized that the study population of the twin registry study is in principle not generalizable to Sweden. Only twins born in Sweden are included in this registry. Not including migrants, who are more likely to receive disability pensions due to mental health diagnoses than non-migrants, would probably have influenced the results of their study, especially since migration status might also be associated with self-employment [51,52]. Compared with the twin registry study, Tiikkaja’s national registry study of hospitalizations included the entire Swedish population, regardless of possible migration background [30].

Unlike the two Swedish studies, the third longitudinal study, a study from South Korea, showed no difference between self-employed and employed persons. This could be due to the fact that Jang et al. only looked at depressive symptoms as an outcome while the Swedish study included all major mental disorders [31]. However, it is also conceivable that the definition of depressive symptoms as an outcome in the South Korean study resulted in no difference between the two groups. Depressive symptoms were assessed with an established instrument, the Center for Epidemiologic Studies Depression Scale (CES-D 11), but they were analyzed for the present study with a much lower cut-off than recommended in the validated version of the CES-D [53,54]. With a too low-threshold cut-off and thus the recording of rather mild and moderate depressive symptoms, differences in the severe forms of depression, which may have led to hospital admissions, were no longer discernible. Other mental disorders were not examined. Furthermore, the observation period of 6 years in the South-Korean study may have been too short to detect long-term mental health effects. In comparison, the two Swedish studies were much longer with 15- and 25-year follow-up assessments, respectively [16,30,31]. It is also possible that the Korean study did not find an association between self-employment and depression or anxiety, because it did not differentiate between white- and blue-collar employees. However, future follow-up assessments of this South Korean longitudinal study considering different employment categories, and assessing other mental disorders as well may lead to findings that might be more comparable with those from the national Swedish register study by Tiikkaja et al. [30].

In general, more longitudinal studies examining the influence of self-employment on mental disorders in different settings are needed as this study design is clearly superior to cross-sectional designs. Cross-sectional studies assess the exposure (e.g., employment status, or other potentially influential factors) and the outcome (e.g., mental disorders) only at one point in time. The validity of retrospectively collected information on the beginning and severity of exposure and/or outcome is likely to be limited by recall or other information bias. Thus, in cross-sectional studies it is challenging to assess clearly if the exposure started before the outcome or vice versa. On the contrary, the strength of longitudinal (i.e., cohort) studies is the prospective collection of data that can be regarded as more valid information. Cohort studies allow judging the temporal cause of both the exposure(s) as well as the onset and development of a new disease and its severity. In case of assessing the employment status in population-based studies over years or decades, a cohort study will also allow to assess more validly possible changes between self-employment and employment. In other words, longitudinal studies would much better allow examining whether the employment status may cause mental disorders or whether mental disorders may cause or force individuals to become self-employed, because employed positions are more difficult to obtain or to keep.

#### 4.2.2. Cross-Sectional Studies

The 23 cross-sectional studies of good quality that we included in our systematic evaluation showed a rather heterogeneous picture in terms of study designs and outcomes examined. The difference between the older and more recent European studies may be related to changes in the social security system of some European countries in the last 10–20 years. These changes include measures that provide better coverage for accidents at work, disability, and unemployment for the self-employed [26]. In addition, since 2005, the self-employed in Europe have benefited from more favorable tax and social security conditions and have had to overcome fewer bureaucratic hurdles than before [48,55]. From 2005 to 2008, European self-employed workers were subsidized to the tune of EUR 750 million [48]. Since 2003, Germany has had subsidy programs for start-ups (“Ich-AG”) to facilitate the path to self-employment [56]. Effects of these subsidies may have led to improved living conditions, less work-related stress, and lower prevalence of mental illness among the self-employed in recent years. However, there are no comparisons within the same European countries over the past 20 years that specifically examined the impact of these programs on the mental health of the self-employed. The German study by Stahmeyer et al. is an exception. They evaluated physician-diagnosed depression among members of one of the largest German statutory health insurance companies. The results showed that the prevalence of depression in the overall population increased from 2006 to 2015. However, the increase over this period was much smaller among the self-employed, who now appear to be less affected by depression compared with employees than in the initial analysis with data from 2006 [15].

In contrast to Europe, the Asian studies, all from South Korea and published from 2016 to 2019, showed that the self-employed were more frequently affected by depression and self-rated poor general mental health than employees [17,18,39]. Unlike Europe, which has compulsory social insurance for all workers (self-employed and salaried), self-employed workers in South Korea are not required to purchase insurance by law. However, they can voluntarily choose from different social insurances and acquire, for example, coverage for work injury or coverage for unemployment [57,58,59]. Further, employment has become more unstable after the financial crisis in 1997, forcing many waged-workers to retire in their mid-40 s and start self-employment [60,61]. Many waged-workers who entered self-employment after retirement often quit self-employment because they could not overcome competition from other self-employed and large corporations. This may be have contributed to the high risk of mental illness among the self-employed in South Korea.

The risk of mental illness among the self-employed may differ from country to country depending on the specific socioeconomic and employment situation. It is important to analyze the mental health risk taking into account this situation in each country. The studies from North/Central America and Australia did not show a better mental health status of the self-employed compared to employees. However, the different socioeconomic conditions for the self-employed in different countries need to be considered. In Central America, there is generally no social insurance for the self-employed [41]. Furthermore, in low-income countries, the self-employed appear to have a significantly lower average income than employees [62]. Since income is a strong motivator for entrepreneurs, the goal of maximizing profits in this group of individuals often comes at the expense of health [11,63]. However, health risks for the self-employed also exist in economically stronger countries, such as in North America. For example, in the United States, twice as many self-employed individuals are uninsured compared to employees. Programs, such as “Affordable Care Act” (since 2014) are intended to mitigate this discrepancy in recent years [64]. However, the positive effects of these offerings were likely not yet incorporated in the studies included in the present review (from 2013 to 2016). Despite relatively good income protection for older people, only 28% of the younger unemployed individuals receive unemployment benefits in North America. In comparison, 64% of the unemployed in Western Europe receive government assistance. In Australia, the self-employed are less likely to be covered by health and social insurance than employees, as the employer often bears the cost of contributions, whereas the self-employed must pay them out of their own pocket [65,66]. Furthermore, the self-employed receive the fewest health insurance benefits in Australia compared to employees [65].

However, comparisons of international studies should be made with caution also due to methodological reasons. Despite overall good methodological quality, some of the included cross-sectional studies did not adjust their results for potential confounders, especially socioeconomic factors. These should be adequately accounted for in population-based observational studies because they correlate with the incidence of mental illness, such as low income [67]. However, cross-sectional study designs do not allow for concluding if the observed mental health problems occurred because of or independent of the employment status.

Self-employed sole proprietors showed lower, whereas entrepreneurs with personnel showed higher stress levels than employees [14,45]. These results are in line with Cocker et al. (2013), who described high or very high psychological distress among 37% of owners or managers of small-to-medium enterprises. This study was not included in our review because of the lack of an employed control group [7]. Several studies showed that amongst workers more responsibility at the job may reduce the level of stress experienced by workers [7,12,13,14,33,45,68]. Among some self-employed individuals, in particular entrepreneurs, a greater autonomy (decision-making latitude) may actually decrease stress levels [13,69]. This may explain part of the inconsistent results of the studies examining sole proprietors and small business owners. Future research on mental health effects of the self-employed needs to examine these relevant subgroups among the self-employed further, particularly with regard to the role of sociodemographic factors such as gender, educational level and family status.

In addition to the division of the self-employed into sole proprietors and those who employ others, the self-employed may also be classified into groups according to their professions. A study from Sweden showed clear differences in mortality rates among the self-employed of different industrial sectors. Suicide, for example, was markedly lower among the self-employed in personal services compared with those who worked in agriculture or trade [70]. These results were confirmed by a South Korean study (Yoon JH 2016) that reported a lower prevalence of suicidal ideation among self-employed versus employed sales and service workers [40]. On the contrary, another South Korean study found a higher prevalence of suicidal ideation among the self-employed. The lack of stratifying the results for specific subgroups may explain the differing findings [24]. For medical doctors, a study from Germany found better outcomes in terms of less burnout among the self-employed when compared to employed physicians [36]. Among occupational drivers in a study from Taiwan, the self-employed (usually sole proprietors) showed a higher prevalence of neurotic problems, psychiatric diagnosis and substance misuse compared to employed drivers [71].

In cross-sectional studies reverse causation cannot be excluded, considering that mentally ill persons may be less likely to be in permanent employment and thus rather choose to be self-employed [17,18]. The published evidence though is still scarce. Individuals with ADHD may prefer self-employment and avoid employment situations where good interaction with colleagues and supervisors is needed [72,73]. Similarly, individuals with personality disorders may also display general problems in social interaction, but to our knowledge corresponding studies are lacking. However, ADHD and personality disorder patients also have a significantly increased risk of developing further mental disorders like depression, anxiety and substance use disorders. This could also contribute to the increased prevalence of other mental disorders among the self-employed even in generally good economic conditions [74,75]. Therefore, further studies that will investigate the well-being of the self-employed should collect information on their financial situation, which is an important determinant for mental and physical health regardless of the employment status.

### 4.3. Strengths and Potential Limitations

This has been the first global systematic review about the occurrence of mental illness among the self-employed. Searching for appropriate studies, we did not exclude any language and country. In addition to this comprehensive approach to get a global perspective, we focused on studies that included comparison groups of employees. We assessed the methodological quality, in particular the risk of bias, in each study with a widely used instrument for observational studies (Table S1).

Several potential limitations have to be mentioned. First, although we searched several large medical databases, complemented by hand searching other internet sources and the references of included publications, we may have missed relevant studies if they were published elsewhere. Second, we found that outcomes and employment status categories were too heterogeneous across the included studies to consider conducting meta-analysis. In general, the studies of our systematic review used rather different assessment tools, which may hamper their comparability (Table 2 and Table 3). We identified 21 different outcome measures including several studies, which had used non-validated questionnaires that were newly developed for the specific investigations. Third, the majority of the studies compared the self-employed with employees from different occupational sectors. In many studies, the group of the self-employed was also rather heterogeneous. There was only one study that described the different mental health problems of the self-employed and employees from the same occupation (doctors) [36]. In this respect, the Eurofond and International Labor Organization stated that “wide-scale differences in job quality in all countries are evident, reflecting the different nature of work across sectors, occupations and workplaces” [69]. Fourth, there is no official distinction between independent self-employed workers and persons who economically depend on an employer [76].

Fifth, the international comparability between the included studies may be further hampered due to considerably different working conditions. For example, the decision latitude of workers from South Korea is at least one third less than decision latitude of other countries. Additionally, cognitive demands at work, including “learning new things”, “complex tasks” and “solving unforeseen problems on your own”, are less common in China and the Republic of Korea in contrast to European countries. Workers in European countries also show a lower incidence of “working over 48 hours a week” compared with other countries, while China, the Republic of Korea and Turkey worked the most hours per week [69]. Additionally, sole proprietors (i.e., self-employed individuals without employees), whose proportion in the general population is increasing due to the global demand for labor market flexibility, have a higher risk of entering into a precarious economic situation. The self-employed with employees, on the other hand, face less pressure because they have more financial resources [77,78].

Sixth, for the included studies from across the globe, which were conducted at different times, we were not able to take into account the overall economic situation, whether a period of growth or a recession. An economic crisis may lead to a higher percentage of precarious self-employment and thus increasing the occurrence of mental disorders, whereas a good economic situation could have the opposite effect [26].

### 4.4. Implications and Outlook

This is the first systematic summary of mental health outcomes comparing the self-employed with employees and including all adequate population-based studies. Our work will contribute to a better understanding of the mental health status of sole proprietors and entrepreneurs, a large but very heterogeneous population group. Our results and the identification of current knowledge gaps will inform decision makers and occupational health researchers to plan future research strategies on work-related and other potential risk factors for mental and physical diseases of the self-employed.

Our review, which applies stringent study quality assessments, was strongly needed considering the inconsistent results of previous and recently published studies. However, for most parts of the world, the self-employed are still a largely understudied population in occupational health sciences. Few countries, like Sweden and South Korea, seem to be the exception. In the latter, for instance, a number of representative surveys showed consistently higher prevalence estimates of mental disorders, including depression, among the self-employed, pointing towards the need for public health action and perhaps specific occupational health promotion programs. For other countries, according to the current evidence, measures regarding the prevention of stress and the promotion of general well-being may be beneficial targets to help the self-employed sustain their mental health.

For several mental outcomes, especially in cross-sectional evaluations, we found no significant differences between the self-employed and employees. This may have been due to design or methodological limitations but may also emphasize the need in occupational health research to differentiate better between very specific occupational subgroups of self-employment. 

Due to technical and social changes, the number of persons in self-employment is increasing worldwide, thus requiring more attention from social policy and preventive medicine [26]. Creating further evidence may help adapting social security laws and preventive offers by public health institutions and medical insurance companies. On an individual level, further evidence will support general practitioners offering their self-employed patients’ specific information on mental health promotion strategies. The current pandemic has shown how vulnerable sole proprietors in many branches are and how in particular the female self-employed were affected [79].

## 5. Conclusions

Despite a comprehensive search for population-based studies on the mental health of the self-employed, we were able to identify only a relatively small number of studies of good quality. The three longitudinal studies showed partly inconsistent results. Evidence from the two longitudinal studies in Europe (Sweden) showed that the self-employed had a lower incidence of mental disorders compared to blue-collar workers, but inconsistent results for the comparison with white-collar workers. The Asian longitudinal study (from South Korea) showed no difference in the incidence of mental disorders between self-employed and employees, not differentiating the latter by blue- and white-collar workers.

Among the cross-sectional studies, those from Asia (all South Korea) showed a higher prevalence of mental disorders among the self-employed compared to employees, whereas those from America, Australia and Europe (the latter since 2010) found lower or similar prevalence estimates of mental disorders among the self-employed compared to employees.

As a basis for targeted prevention strategies, further longitudinal population-based studies in different settings of self-employment are strongly required to understand the development of mental health disorders for specific self-employment categories such as sole proprietors, small entrepreneurs, farmers, family businesses and others.

## Figures and Tables

**Table 1:** Quality assessment by Hoy et al. of the included studies with good quality.

Risk of Bias Items	Quality Assessment of The Included Studies 0 = Yes (Low Risk of Bias) 1 = No (High Risk of Bias)																									
	Tiikkaja 2013 [30]	Samuelsson 2012b [16]	Jang 2015 [31]	Stahmeyer 2019 [15]	Combois 2017 [32]	Rosta 2014 [33]	Lindström 2012 [34]	Samuelsson 2012a [35]	Heinke 2011 [36]	Stephan 2010 [25]	Saarni 2007 [37]	Muntaner 2003 [38]	Gehring 2001 [13]	Benavides 2000 [14]	Kim 2019 [17]	Park 2019 [39]	Kim 2016 [18]	Yoon 2016 [40]	Yoon 2015 [24]	Cardon 2015 [11]	Lopez-Ruiz 2015 [41]	Rietveld 2015 [42]	Merchant 2014 [43]	Yoon 2013 [44]	Hessels 2016 [45]	Parslow 2014 [46]
1. The study’s target population was a close representation of the national population.	0	1	0	1	0	0	0	1	0	0	0	1	0	0	0	0	0	1	0	1	0	0	1	0	0	1
2. The sampling frame was a true or close representation of the target population.	0	1	0	0	1	0	0	0	0	0	0	0	0	0	0	0	0	0	0	0	0	0	0	0	0	0
3. A census was undertaken, OR, some form of random selection was used to select the sample.	0	0	1	0	0	1	0	0	0	0	0	0	0	1	0	0	0	0	0	1	1	0	0	1	0	0
4. Was the likelihood of non-response bias minimal (response rate ≥ 75%)?	0	0	1	0	0	1	1	0	1	1	0	1	1	1	1	1	1	1	1	0	1	1	1	1	1	1
5. All data were collected directly from the subjects.	0	1	0	0	0	0	0	0	0	0	0	0	0	0	1	0	0	0	0	0	0	0	0	0	0	0
6. An acceptable case definition was used.	0	0	0	0	0	0	0	0	0	0	1	0	0	0	0	0	1	0	0	1	0	0	1	0	0	0
7. The study instrument had been shown to have reliability and validity.	0	0	0	0	0	0	0	0	0	0	0	0	1	1	0	0	1	1	1	0	0	0	0	0	1	0
8. The same mode of data collection was used for all subjects.	0	0	0	0	0	0	0	0	0	0	0	0	0	0	0	0	0	0	0	0	0	0	0	0	0	0
9. Were the numerator(s) and denominator(s) for the parameter of interest appropriate? (* = not applicable)	*	0	*	1	*	*	*	*	0	*	*	0	0	0	1	1	0	0	*	0	0	*	0	*	*	*
Summary 0–3: low risk of bias for studies that reported prevalence estimates; 0–2: low risk of bias for studies that did not report prevalence estimates (assessed by question 9)	0	3	2	2	1	2	1	1	1	1	1	2	2	3	3	2	3	3	2	3	2	2	3	2	2	2

**Table 2:** Basic characteristics and results of **longitudinal observational** studies of good quality on mental disorders comparing self-employed (s-empl) vs. employees (empl).

Author, Publication Year [Reference]	Study Design, Country, Recruitment Year	Baseline Number, Age, Females	Follow-Up Length, Last Response	Source Population	Mental Health Outcome Definition	Results
**EUROPE**						
**Tiikkaja 2013** **[30]**	Historic cohort study, Sweden 1980	*n* = 1,016,276 range 21–31 y 38%	25 y, 99% (census 1980), 98% (census 1990)	Censuses data from 1980 & 1990, National Patient and other registers used to identify all working individuals without psychiatric diagnoses in Sweden before 1980	**First hospitalization** for **a severe psychiatric disorder such as:** schizophrenia, alcoholism, drug dependency, affective psychosis, neurosis, or personality disorder	**Severe psychiatric disorder (first hospitalization)** **aRR** Empl (high white-collar): 1.0 (ref.-categ.) Empl (low white-collar): 1.34 (95%-CI 1.29–1.40) **S-empl: 1.55 (95%-CI 1.47–1.64)** Empl (high blue-collar): 1.60 (95%-CI 1.54–1.67) Empl (low blue-collar): 1.98 (95%-CI 1.91–2.05) Incidence rates Empl (high white-collar): 90/100,000 person-years Empl (low white-collar): 121/100,000 person-years **S-empl: 152/100,000 person-years** Empl (high blue-collar): 165/100,000 person-years Empl (low blue-collar): 189/100,000 person-years
**Samuelsson 2012b** **[16]**	Register cohort study, Sweden 1993	*n* = 52,509 mean 47.8 y 51%	15 y, 95%	Swedish Twin Registry, Swedish National Social Insurance Agency, Causes of Death Registry	**Disability pension** due to **a mental diagnosis:** ICD10: F00–F99	**Mental illness (reason for disability pension)** HR adjusted for age only Empl (high white-collar): 0.97 (95%-CI 0.85–1.17) Empl (middle white-collar): 1.0 (ref.-categ.) Empl (low white-collar): 0.89 (95%-CI 0.73–1.09) **S-empl: 0.48 (95%-CI 0.35–0.65)** Empl (skilled blue-collar): 0.71 (95%-CI 0.57–0.87) Empl (unskilled blue-collar):1.10 (95%-CI 0.93–1.29) Incidence: males 3.3% vs. 12.4%, females 1.5% vs. 19.8%
**ASIA**						
**Jang 2015** **[31]**	Cohort study South Korea, 2007	*n* = 3981 range 20 y–59 y 45%	6 y, 77–79%	Korean Welfare Panel Study (KOWEPS) 2007–2013: nationally representative sample	**The Center for Epidemiological Studies Depression Scale** (South Korean version)	**S-empl vs. full time permanently empl (ref.-categ.):** **Severe depressive symptoms** **Males aOR: 1.09 (95%-CI 0.71–1.66)** Incidence in males: 1.8% vs. 1.5% **Females aOR: 1.21 (95%-CI 0.69–2.11)** Incidence in females: 3.0% vs. 1.9%

**Table 3:** Basic characteristics of **cross-sectional** studies of good quality on mental disorders comparing self-employed (s-empl) vs. employees (empl).

Author, Publication Date [Reference]	Country/Region of Study	Sample size, Female	Age [Mean (SD) or Range]	Source Population	Occupational Groups	Disease Outcomes: Assessment Tools
**EUROPE**						
**Stahmeyer 2019** **[15]**	Germany/Lower Saxony (federal state in Northern Germany)	1,838,965 (2015), 1,667,495 (2006), 52.3%	43.8 (19.2) y (2015), n.r. (2006)	Largest statutory health insurance company in federal state Lower Saxony: routine care data	s-empl vs. empl	**Depressive episode/Depressive disease (physician’s diagnosis):** ICD-10: F32, F33
**Cambois 2017** **[32]**	France	7537, 53%	45–74 y	French Population Survey 2006	s-empl (farm/business owner) vs. empl (low-skilled clerical) vs. empl (unskilled clerical) vs. empl (manual workers)	**Depressive symptoms:** Mini International Neuropsychiatric Interview (MINI)
**Rosta 2014** **[33]**	Norway	834, 23.8% (s-empl) 43.0% (empl)	29–67 y	Research Institute of the Norwegian Medical Association: Active Norwegian doctors	s-empl (doctors) vs. empl (doctors)	**Psychological stress at work:** short form of the effort-reward questionnaire (ERI)
**Lindström 2012** **[34]**	Sweden	28,198, 54.9%	18–80 y	Public health survey in Skane 2008: randomly selected from the official population registers of people living in Skane	s-empl vs. empl (high non-manual worker)	**Self-rated mental health:** 12-Item General Health Questionnaire (GHQ-12)
**Samuelsson 2012a** **[35]**	Sweden	877, 46%	42 y	School leavers in Lulea City	s-empl vs. empl (temporary) vs. empl (permanent)	**Exhaustion:** Subscale of the Swedish Maslach Burnout Inventory
**Heinke 2011** **[36]**	Germany	3541, 32.7%	44 (8.5) y	Professional Association of German Anaesthetists and German Society for Anaesthesiology and Intensive Care (DGAI)	s-empl (anaesthetists) vs. empl (anaesthetists)	**Burn-Out-Syndrome:** BOS-Sum-Score (Copenhagen Psychosocial Questionnaire CAPSOQ)
**Stephan 2010** **[25]**	Germany	298, 26.5%	46.2 (8.7) y	Matched case-control design with participants from German National Health Survey 1998	s-empl vs. empl	**1. Somatic diseases:** ICD-10 **2. Mental disorders:** DIA-X-Munich Composite International Diagnostic Interview (DIA-X-M-CIDI)
**Saarni 2007** **[37]**	Finland	5184, 50%	46 (38–53) y	Health 2000 survey: two stage stratified cluster sampling	s-empl (without personnel) vs. s-empl (with personnel) vs. empl	**1. Mental function, depression, distress:** 15D **2. Anxiety and or depression:** EQ-5D
**Muntaner 2003** **[38]**	Spain	4219, 44.4%	16–64 y	Non-institutionalised population of Barcelona City in 2000	s-empl (small enterprise) vs. empl (organizational non-managerial supervisors)	**Self-rated mental health:** 12-Item General Health Questionnaire (GHQ-12)
**Gehring 2001** **[13]**	Switzerland	1200, 60%	15–74 y	Random sample of native speakers drawn from the Swiss telephone directory	s-empl vs. empl (with low responsibilities)	**Perceived stress:** “yes/no”—question
**Benavides 2000** **[14]**	Spain	15,146, n.r.	>15 y	Multi-stage random sample of the working population of 15 EU-countries (1000 cases per country)	s-empl (without personnel) vs. s-empl (with personnel) vs. empl (permanent)	**Stress, fatigue:** New questionnaire developed for the study
**ASIA**						
**Kim 2019** **[17]**	South Korea	32,630, 40.5%	20–59 y	Third Korean Working conditions Survey (KWCS, 2011)	s-empl (small business owners) vs. empl (full-time)	**Mental well-being:** WHO-5 well-being index
**Park 2019** **[39]**	South Korea	44,266, 48.1%	n.r.	Fourth Korean Working conditions Survey (KWCS, 2014)	s-empl vs. empl (mental vs. emotional vs. physical work)	**Mental well-being:** WHO-5 well-being index
**Kim 2016** **[18]**	South Korea	48,850, 42.2%	45.8 (13.1) y	Third Korean Working conditions Survey (KWCS, 2011)	s-empl vs. empl (full-time)	**Depressive and anxiety disorder:** “yes/no”-question
**Yoon 2016** **[40]**	South Korea	1995, 58.7%	n.r.	4th Korea National Health and Nutrition Examination Survey (KNHANES)	s-empl vs. empl	**Suicidal ideation:** “yes/no”-question
**Yoon 2015** **[24]**	South Korea	67,471, 35.5%	n.r.	2008 CHS conducted by the Korea Center for Disease Control and Prevention (KCDC)	s-empl vs. empl	**Suicidal ideation:** “yes/no”-question
**NORTH and CENTRAL AMERICA**						
**Cardon 2015** **[11]**	USA (New York, Indiana)	1376, n.r.	25–74 y	NHANES I Epidemiologic Follow-up Study (NHEFS)	s-empl vs. empl	**Stress:** (a) General Well-Being Schedule, (b) Centre for Epidemiological Studies-Depression Scale (CES-D)
**Lopez-Ruiz 2015** **[41]**	Central America (Authors: Spain)	8823, 48.5%	>18 y	First Central American Survey of Working Conditions and Health (Spanish acronym: ECCTS)	s-empl (without personnel) vs. s-empl (<5 employees) vs. empl	**Mental health:** 12-Item General Health Questionnaire (GHQ-12)
**Rietveld 2015** **[42]**	USA (Authors: Netherlands)	30,671, 58%	56.7 (5.0) y (s-empl) 55.9 (5.2) y (empl)	US Health and Retirement study (HRS): representative sample of Americans over 50 years	s-empl vs. empl	**Depression:** Center for Epidemiologic Studies Depression Scale (CES-D) from 0 (absence of depression symptoms) to 8 (presence of all measured depression symptoms)
**Merchant 2014** **[43]**	USA (Iowa)	1271, 53.4% (s-empl) 66.2% (empl)	18–68 y	2010 Iowa Voter Registration database	s-empl vs. empl (organizational)	**Quality of Life:** Real Iowans Health Survey Questionnaire
**Yoon 2013** **[44]**	USA (Oregon)	13,435, n.r.	45.1 (10.8) y (s-empl) 39.6 (12.2) y (empl)	2007 Survey MEPS	s-empl vs. empl	**1. Mental health:** Mental Health Composite Score, **2. Stress:** Kessler Psychological Distress Scale (K10)
**AUSTRALIA**						
**Hessels 2016** **[45]**	Australia	68,264, 63% (s-empl) 50% (empl)	18–64 y	HILDA (Household, Income and Labour Dynamics in Australia) survey: working individuals	s-empl (without employees) vs. s-empl (with employees) vs. empl	**Work-related stress:** 2-item 7-point Likert scale
**Parslow 2014** **[46]**	Australia (Author: United Kingdom)	2530, n.r.	40–44 y	Australian Electoral Rolls for Canberra in the Australian Capital Territory and adjacent town of Queanbeyan in New South Wales	s-empl (without personnel) vs. s-empl (with personnel) vs. empl (organizational)	**Depression and/or anxiety:** Goldberg’s depression and anxiety scale

SD = standard deviation, y = years, vs. = versus, n.r. = not reported.

**Table 4:** Overview of mental health outcomes among self-employed compared to employed persons in longitudinal and cross-sectional studies.

Study Design	Author/ Publication Date [Reference]	Mental Illness (at Least One)		Depression or Suicidal Thoughts		Anxiety		Stress or Exhaustion or Burnout		Self-Rated Poor General Mental Health		
**Longitudinal**	**EUROPE**											
	Tiikkaja 2013 [30]	**vs blue-collar**	**vs white- collar**									
	Samuelsson 2012b [16]	**vs** **blue -collar**	**vs white-collar**									
	**ASIA**											
	Jang 2015 [31]											
**Cross-sectional**	**EUROPE**											
	Stahmeyer 2019 (data 2015) [15]			female	male							
	Cambois 2017 [32]			female	male							
	Rosta 2014 [33]											
	Lindström 2012 [34]											
	Samuelsson 2012a [35]							Exhaust. (female)	Exhaust. (male)			
	Heinke 2011 [36]							Burnout				
	Stephan 2010 [25]											
	Saarni 2007 [37]									sole	non-sole	farm
	Stahmeyer 2019 (data 2006) [15]			female	male							
	Muntaner 2003 [38]											
	Gehring 2001 [13]											
	Benavides 2000 [14]							sole	non- sole			
	**ASIA**											
	Kim 2019 [17]											
	Park 2019 [39]											
	Kim 2016 [18]											
	Yoon 2016 [40]			Suicidal thoughts								
	Yoon 2015 [24]			Suicidal thoughts								
	**NORTH and CENTRAL AMERICA**											
	Cardon 2015 [11]											
	Lopez-Ruiz 2015 [41]											
	Rietveld 2015 [42]											
	Merchant 2014 [43]											
	Yoon 2013 [44]											
	**AUSTRALIA**											
	Hessels 2016 [45]							**sole**	**non-** **sole**			
	Parslow 2014 [46]			sole	non- sole	sole	non- sole					

The self-employed showed: 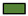 = significantly lower occurrence; 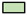 = lower occurrence; 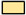 = no difference; 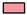 = higher occurrence; 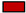 = significantly higher occurrence, compared to the employed. Self-employment subgroups: “sole” = sole proprietorship (vs the employed); “non-sole” = employing others (vs the employed); “farm” = farm workers.

**Table 5:** **Mental illness, at least one**—results of cross-sectional studies of good quality on mental disorders comparing self-employed (s-empl) vs. employees (empl).

Author, Publication Date [Reference]	Results
Stephan 2010 [25]	**1. Mental disorders** s-empl vs. empl (ref.): OR 0.58 (95%-CI 0.36–0.93) * 2. Somatoform disorders s-empl vs. empl (ref.): OR 0.29 (95%-CI 0.11–0.75) * 3. Affective disorders s-empl vs. empl (ref.): OR 0.96 (95%-CI 0.47–1.94) **4. Substance abuse/dependence** s-empl vs. empl (ref.): OR 1.08 (95%-CI 0.35–3.31)

* *p* < 0.050, vs. = versus, ref. = reference, CI = confidence interval, OR = odds ratio.

**Table 6:** **Depression**—results of cross-sectional studies of good quality on mental disorders comparing self-employed (s-empl) vs. employees (empl).

Author, Publication Date [Reference]	Results
Kim 2019 [17]	**Depression** s-empl vs. empl (ref.): 15.9% vs. 12.1%, OR 1.20 (95%-CI 1.09–1.32) *
Stahmeyer 2019 [15]	**Depression** s-empl vs. empl: Male (2006): 4.5% vs. 3.8%; Female (2006): 11.1% vs. 10.9% Male (2015): 5.1% vs. 5.9%; Female (2015): 13.6% vs. 15.2%
Cambois 2017 [32]	**Major depressive episodes** s-empl vs. empl (ref.): male: OR 0.47; female: OR 1.76
Rietveld 2015 [42]	**Depression symptoms** s-empl vs. empl [0 (absence of depression symptoms) to 8 (presence of all measured depression symptoms)] Mean 0.56 (SD 0.50) vs. Mean 0.54 (SD 0.50)
Parslow 2014 [46]	**Depression** s-empl (without personnel) vs. s-empl (with personnel) vs. empl (ref.): male [Mean(β)]: 2.09 (−0.261) vs. 2.27 (−0.295) vs. 2.21, female [Mean(β)]: 2.61 (0.271) vs. 2.5 (0.216) vs. 2.44

* *p* < 0.050, vs. = versus, ref. = reference, CI = confidence interval, aOR = adjusted odds ratio, OR = odds ratio, SD = standard deviation.

**Table 7:** **Suicidal thoughts**—results of cross-sectional studies of good quality on mental disorders comparing self-employed (s-empl) vs. employees (empl).

Author, Publication Date [Reference]	Results
Yoon J-H 2016 [40]	**Suicidal ideation** s-empl vs. empl: male: 8.7% vs. 10.9% female: 18.1% vs. 22.1%
Yoon C-G 2015 [24]	**Suicidal ideation** s-empl vs. empl: male: 5.4% vs. 3.7% * female: 11.3% vs. 8.3% *

* *p* < 0.050, vs. = versus, ref. = reference.

**Table 8:** **Anxiety**—results of cross-sectional studies of good quality on mental disorders comparing self-employed (s-empl) vs. employees (empl).

Author, Publication Date [Reference]	Results
Stephan 2010 [25]	**Anxiety disorders** s-empl vs. empl (ref.): OR 0.92 (95%-CI 0.43–1.97)
Parslow 2004 [46]	**Anxiety** s-empl (without personnel) vs. s-empl (with personnel) vs. empl (ref.): male [Mean(β)]: 3.25 (−0.276) vs. 2.89 (−0.310) vs. 3.21, female [Mean(β)]: 3.84 (0.105) vs. 3.63 (0.069) vs. 3.60

* *p* < 0.050, vs. = versus, ref. = reference, CI = confidence interval, aOR = adjusted odds ratio.

**Table 9:** **Stress, burnout, exhaustion**—results of cross-sectional studies of good quality on mental disorders comparing self-employed (s-empl) vs. employees (empl).

Author, Publication Date [Reference]	Results
Hessels 2016 [45]	**Work-related** stress s-empl (without personnel) and s-empl (with personnel) vs. empl [Mean(SD); 7-point Likert scale (1 = no stress; 7 = extreme stress)]: 2.49 (1.38) * and 2.91 (1.47) vs. 2.78 (1.45)
Cardon 2015 [11]	**Stress level** (subjective) s-empl vs. empl (ref.) (data from 1987): 0.136
Rosta 2014 [33]	High levels of **psychosocial work stress** s-empl vs. empl: 11.4% vs. 24.3% *
Yoon 2013 [44]	**Psychological distress** s-empl vs. empl (ref.) [Mean(SD); logarithm of Kessler Psychological Distress Scale (10 = no distress; 50 = extreme distress)]: 0.97 (0.80) vs. 0.99 (0.83)
Samuelsson 2012a [35]	**Exhaustion** s-empl vs. empl (temporary) vs. empl (permanent) [Mean; subscale of the Swedish Maslach Burnout Inventory (1 = no exhaustion; 6 = extreme exhaustion)]: male: 1.95 vs. 2.06 vs. 2.09 female: 2.28 vs. 2.26 vs. 2.32
Heinke 2011 [36]	**Burn-Out-Symptom** s-empl (physician) vs. empl (physician) [Mean(SD); Personal Burnout Scale of the Copenhagen Burnout Inventory (12 = no burn-out symptom; 60 = extreme burn-out symptom)]: 38.1 (20.5) * vs. 43.0 (19.2) **Burn-Out-Symptom** s-empl (free-lance/established doctor) vs. empl (private/public/university hospital): 30.5%/33.2% vs. 40.8%/40.9%/42.8%
Gehring 2001 [13]	**Perceived stress** s-empl vs. empl (ref.): 47.1% vs. 35.7%; OR 2.10 (95%-CI 1.30–3.41) *
Benavides 2000 [14]	**Stress** s-empl without and with personnel vs. empl (full-time, ref.): 32.3% and 34.3% * vs. 29.5%

* *p* < 0.050, vs. = versus, ref. = reference, CI = confidence interval, OR = odds ratio, SD = standard deviation.

**Table 10:** **Self-rated poor general mental health**—results of cross-sectional studies of good quality on mental disorders comparing self-employed (s-empl) vs. employees (empl).

Author, Publication Date [Reference]	Results
Park 2019 [39]	**Self-rated poor general mental health** s-empl vs. empl: male non-manual workers with cognitive demands: 36.0% vs. 31.0% *, female non-manual workers with cognitive demands: 33.1% vs. 31.0% male service and sales workers: 44.0% vs. 36.0% *, female service and sales workers: 45.7% vs. 40.3% * male manual workers: 53.2% vs. 47.0% *, female manual workers: 59.9% vs. 52.9% *
Kim 2016 [18]	**Depression and anxiety disorder** s-empl vs. empl: 1.69% vs. 1.25% *
Lopez-Ruiz 2015 [41]	**Self-rated poor general mental health** s-empl (without personnel) and s-empl (<5 employees) vs. empl (ref.) (women/men): 34%/29%; 28%/27%; 27%/24%; aOR 1.27 (95%-CI 1.05–1.54) and aOR 1.33 (1.07–1.65) vs. OR 1.00 (ref.)
Merchant 2014 [43]	**No days with mental health problems (last 4 months)** s-empl vs. empl (organizational): 68.1% vs. 65.3% **No days with sad feelings (last 4 months)** s-empl vs. empl (organizational): 67.4% vs. 64.9% **No days with worries/tension (last 4 months)** s-empl vs. empl (organizational): 43.5% vs. 39.9%
Yoon 2013 [44]	**Perceived mental health** s-empl vs. empl (ref.) [Mean(SD); 5-point Likert scale (1 = worst mental health; 5 = good mental health)]: 4.11 (0.90) vs. 4.04 (0.20); effect size (aSE) 0.176 (0.097)
Lindström 2012 [34]	**Self-rated poor general mental health** s-empl vs. empl (high-level white-collar = ref.): men: 11.5% vs. 13.1%; aOR 0.75 (95%-CI 0.56–0.99) women: 15.0% vs. 16.7%; aOR 0.77 (95%-CI 0.57–1.06)
Saarni 2007 [37]	**Self-reported anxiety and/or depression** s-empl (without personnel), s-empl (with personnel) and farmers vs. empl (ref.): OR 1.39 (95%-CI 0.75–0.56), OR 0.67 (0.35–1.28) and OR 1.05 (0.51–2.16) vs. OR 1.00 (ref.)
Muntaner 2003 [38]	**Self-rated poor general mental health** s-empl (of small enterprise) vs. empl (skilled supervisors = ref.): men: 10.1% vs. 6.6%; aOR 1.98 (95%-CI 0.83–4.73) women: 15.0% vs. 8.2%; aOR 2.51 (95%-CI 0.88–7.17)

* *p* < 0.050, vs. = versus, ref. = reference, CI = confidence interval, aOR = adjusted odds ratio, HR = hazard ratio, OR = odds ratio, aSE = adjusted standard error.

## Data Availability

Not applicable since we only evaluated data that has been already published.

## Supplementary Materials

The following are available online at https://www.mdpi.com/article/10.3390/ijerph18168617/s1, Table S1: Quality assessment checklist for prevalence studies (adapted from Hoy et al.), Table S2: Basic characteristics of longitudinal and cross-sectional studies with moderate quality on mental disorders comparing self-employed (s-empl) with employees (empl), Table S3. Results of cross-sectional studies on mental health of moderate or poor quality comparing the self-employed (s-empl) with employees (empl), Table S4. Overview of mental health outcomes among the self-employed compared to employed persons in longitudinal and cross-sectional observational studies with moderate or poor quality.

## References

[1] International Labour Organization (2020). ILOSTAT Database. https://data.worldbank.org/indicator/SL.EMP.SELF.ZS.

[2] OECD (2020). Self-Employment-Rate (indicator)//Employment. https://data.oecd.org/emp/self-employment-rate.htm.

[3] Schonfeld I.S., Mazzola J.J. (2015). A qualitative study of stress in individuals self-employed in solo businesses. J. Occup. Health Psychol..

[4] Sewdas R., Tamminga S.J., Boot C.R.L., van den Heuvel S.G., de Boer A.G., van der Beek A.J. (2018). Differences in self-rated health and work ability between self-employed workers and employees: Results from a prospective cohort study in the Netherlands. PLoS ONE.

[5] Oren L. (2012). Job stress and coping: Self-employed versus organizationally employed professionals. Stress Health J. Int. Soc. Investig. Stress.

[6] Aronsson G. (2000). Sick but yet at work. An empirical study of sickness presenteeism. J. Epidemiol. Community Health.

[7] Cocker F., Martin A., Scott J., Venn A., Sanderson K. (2013). Psychological distress, related work attendance, and productivity loss in small-to-medium enterprise owner/managers. Int. J. Environ. Res. Public Health.

[8] Leignel S., Schuster J.-P., Hoertel N., Poulain X., Limosin F. (2014). Mental health and substance use among self-employed lawyers and pharmacists. Occup. Med..

[9] Chin B., Slutsky J., Raye J., Creswell J.D. (2019). Mindfulness Training Reduces Stress At Work: A Randomized Controlled Trial. Mindfulness.

[10] Plaisier I., de Graaf R., de Bruijn J., Smit J., van Dyck R., Beekman A., Penninx B. (2012). Depressive and anxiety disorders on-the-job: The importance of job characteristics for good work functioning in persons with depressive and anxiety disorders. Psychiatry Res..

[11] Cardon M.S., Patel P.C. (2015). Is Stress Worth it? Stress-Related Health and Wealth Trade-Offs for Entrepreneurs. Appl. Psychol..

[12] Grégoris M., Deschamps F., Salles J., Sanchez S. (2017). Health assessment of self-employed in the food service industry. Int. J. Occup. Environ. Health.

[13] Gehring T.M., Aubert L., Padlina O., Martin-Diener E., Somaini B. (2001). Perceived stress and health-related outcomes in a Swiss population sample. Swiss J. Psychol..

[14] Benavides F.G., Benach J., Diez-Roux A.V., Roman C. (2000). How do types of employment relate to health indicators? Findings from the Second European Survey on Working Conditions. J. Epidemiol Community Health.

[15] Stahmeyer J.T., Kuhlmann K., Eberhard S. (2019). Die Häufigkeit von Depressionsdiagnosen nach Versichertengruppen im Zeitverlauf—Eine Routinedatenanalyse der Jahre 2006–2015. Psychother. Psychosom. Med. Psychol..

[16] Samuelsson Å., Alexanderson K., Ropponen A., Lichtenstein P., Svedberg P. (2012). Incidence of disability pension and associations with socio-demographic factors in a Swedish twin cohort. Soc. Psychiatry Psychiatr. Epidemiol..

[17] Kim H.-J., Min J.-Y., Min K.-B. (2019). Physical and mental health problems of self-employed small business owners in South Korea. Am. J. Ind. Med..

[18] Kim T., Kang M.-Y., Yoo M.-S., Lee D., Hong Y.-C. (2016). Computer use at work is associated with self-reported depressive and anxiety disorder. Ann. Occup. Environ. Med..

[19] Sakisaka K. (2018). Identification of high risk groups with shorter survival times after onset of the main reason for suicide: Findings from interviews with the bereaved in Japan. BMC Res. Notes.

[20] Mofidi N., Ghazinour M., Araste M., Jacobsson L., Richter J. (2008). General mental health, quality of life and suicide-related attitudes among Kurdish people in Iran. Int. J. Soc. Psychiatry.

[21] Hounsome B., Edwards R.T., Hounsome N., Edwards-Jones G. (2012). Psychological morbidity of farmers and non-farming population: Results from a UK survey. Community Ment. Health J..

[22] Kameyama A., Matsumoto T., Katsumata Y., Akazawa M., Kitani M., Hirokawa S., Takeshima T. (2011). Psychosocial and psychiatric aspects of suicide completers with unmanageable debt: A psychological autopsy study. Psychiatry Clin. Neurosci..

[23] Min J.-Y., Kim H., Park S.-G., Hwang S.H., Min K.-B. (2019). Differences in suicidal behaviors between self-employed and standardly employed workers. Am. J. Ind. Med..

[24] Yoon C.-G., Bae K.-J., Kang M.-Y., Yoon J.-H. (2015). Is suicidal ideation linked to working hours and shift work in Korea?. J. Occup. Health.

[25] Stephan U., Roesler U. (2010). Health of entrepreneurs versus employees in a national representative sample. J. Occup. Organ. Psychol..

[26] International Labour Office (2017). Universal Social Protection to Achieve the Sustainable Development Goals.

[27] Higgins J., Thomas J. (2019). Cochrane Handbook for Systematic Reviews of Interventions, Version 6.

[28] Moher D., Liberati A., Tetzlaff J., Altman D.G. Preferred Reporting Items for Systematic Reviews and Meta-Analyses: The PRISMA Statement. http://www.prisma-statement.org/.

[29] Hoy D., Brooks P., Woolf A., Blyth F., March L., Bain C., Baker P., Smith E., Buchbinder R. (2012). Assessing risk of bias in prevalence studies: Modification of an existing tool and evidence of interrater agreement. J. Clin. Epidemiol..

[30] Tiikkaja S., Sandin S., Malki N., Modin B., Sparén P., Hultman C.M. (2013). Social class, social mobility and risk of psychiatric disorder—A population-based longitudinal study. PLoS ONE.

[31] Jang S.-Y., Jang S.-I., Bae H.-C., Shin J., Park E.-C. (2015). Precarious employment and new-onset severe depressive symptoms: A population-based prospective study in South Korea. Scand. J. Work. Environ. Health.

[32] Cambois E., Garrouste C., Pailhé A. (2017). Gender career divide and women’s disadvantage in depressive symptoms and physical limitations in France. SSM Popul. Health.

[33] Rosta J., Tellnes G., Aasland O.G. (2014). Differences in sickness absence between self-employed and employed doctors: A cross-sectional study on national sample of Norwegian doctors in 2010. BMC health Serv. Res..

[34] Lindström M., Ali S.M., Rosvall M. (2012). Socioeconomic status, labour market connection, and self-rated psychological health: The role of social capital and economic stress. Scand. J. Public Health.

[35] Samuelsson Å., Houkes I., Verdonk P., Hammarström A. (2012). Types of employment and their associations with work characteristics and health in Swedish women and men. Scand. J. Public Health.

[36] Heinke W., Dunkel P., Brähler E., Nübling M., Riedel-Heller S., Kaisers U.X. (2011). Burn-out in der Anästhesie und Intensivmedizin: Gibt es ein Problem in Deutschland?. Anaesthesist.

[37] Saarni S.I., Saarni E.S., Saarni H. (2008). Quality of life, work ability, and self employment: A population survey of entrepreneurs, farmers, and salary earners. Occup. Environ. Med..

[38] Muntaner C., Borrell C., Benach J., Pasarín M.I., Fernandez E. (2003). The associations of social class and social stratification with patterns of general and mental health in a Spanish population. Int. J. Epidemiol..

[39] Park J., Han B., Kim Y. (2019). Comparison of occupational health problems of employees and self-employed individuals who work in different fields. Arch. Environ. Occup. Health.

[40] Yoon J.-H., Jeung D., Chang S.-J. (2016). Does High Emotional Demand with Low Job Control Relate to Suicidal Ideation among Service and Sales Workers in Korea?. J. Korean Med. Sci..

[41] López-Ruiz M., Artazcoz L., Martínez J.M., Rojas M., Benavides F.G. (2015). Informal employment and health status in Central America. BMC Public Health.

[42] Rietveld C.A., van Kippersluis H., Thurik A.R. (2015). Self-Employment and Health: Barriers or Benefits?. Health Econ..

[43] Merchant J.A., Kelly K.M., Burmeister L.F., Lozier M.J., Amendola A., Lind D.P., KcKeen A., Slater T., Hall J.L., Rohlman D.S. (2014). Employment status matters: A statewide survey of quality-of-life, prevention behaviors, and absenteeism and presenteeism. J. Occup. Environ. Med..

[44] Yoon J., Bernell S.L. (2013). The effect of self-employment on health, access to care, and health behavior. Health.

[45] Hessels J., Rietveld C.A., van der Zwan P. (2017). Self-employment and work-related stress: The mediating role of job control and job demand. J. Bus. Ventur..

[46] Parslow R.A., Jorm A.F., Christensen H., Rodgers B., Strazdins L., D’Souza R.M. (2004). The associations between work stress and mental health: A comparison of organizationally employed and self-employed workers. Work Stress.

[47] Sevä I.J., Larsson D. (2015). Are the self-employed really that poor? Income poverty and living standard among self-employed in Sweden. Soc. Health Vulnerability.

[48] Europäische Kommission (2010). Bericht des Europäischen Beschäftigungsobservatoriums: Selbstständige Erwerbstätigkeit in Europa 2010.

[49] Andersson L., Nyman C.S., Spak F., Hensing G. (2006). High incidence of disability pensionwith a psychiatric diagnosis in western Sweden. A population-based study from 1980 to 1998. Work.

[50] Andersson L., Wiles N., Lewis G., Brage S., Hensing G. (2007). Can access to psychiatric health care explain regional differences in disability pension with psychiatric disorders?. Soc. Psychiatry Psychiatr. Epidemiol..

[51] Österberg T., Gustafsson B. (2006). Disability pension among immigrants in Sweden. Soc. Sci. Med..

[52] Beckman A., Hakansson A., Rastam L., Lithman T., Merlo J. (2006). The role country of birth plays in receiving disability pensions in relation to patterns of health care utilisation and socioeconomic differences: A multilevel analysis of Malmo, Sweden. BMC Public Health.

[53] Cho M.J., Kim K.H. (1998). Use of the Center for Epidemiologic Studies Depression (CES-D) Scale in Korea. J. Nerv. Ment. Dis..

[54] Lenore Sawyer Radloff (1977). The CES-D Scale: A Self-Report Depression Scale for Research in the General Population. Appl. Psychol. Meas..

[55] Eurofoun (2017). Exploring Self-Employment in the European Union.

[56] § 57 SGB III durch Artikel 1 Nr. 3 Gesetz zur Verbesserung der Eingliederungschancen am Arbeitsmarkt. https://www.sozialgesetzbuch-sgb.de/sgbiii/443.html.

[57] Lee S. (2015). Social Security System of South Korea.

[58] Social Security Administration (2014). Social Security Programs throughout the World: Asia and the Pacific.

[59] Selbstständigkeit. https://www.infobest.eu/de/themengebiete/artikel?tx_infobestfaq_faq%5Baction%5D=list&tx_infobestfaq_faq%5Bcontroller%5D=Article&tx_infobestfaq_faq%5Btag%5D=106&cHash=7631bb277636120b01c384809aaca903.

[60] Self-Employed in Crisis. http://www.koreaherald.com/view.php?ud=20200910000635#.

[61] Kim D.I. (2002). The Korean labor market: The Crisis and After. Int. Monet. Fund.

[62] World Employment and Social Outlook-Trends 2020. http://www.ilo.org.

[63] Benzing C., Chu H.M. (2009). A comparison of the motivations of small business owners in Africa. J. Small Bus. Enterp. Dev..

[64] Reinhardt U.E. (2013). Much ado about nothing: The US Supreme Court’s rules on health reform. Health Econ. Policy Low.

[65] Whiteford P., Heron A. (2018). Dealing with Non-Standard Work in a General Revenue Financed Social Protection System—The Case of Australia: The Future of Social Protection: What Works for Non-Standard Workers?.

[66] Hamilton B.H. (2000). Does entrepreneurship pay? An empirical analyses of the returns to self-employment. J. Polit. Econ..

[67] Schafstein S. (2015). Money and mental Health. Psychiatr. Serv..

[68] Kawakami N., Iwata N., Tanigawa T., Oga H., Araki S., Fujihara S., Kitamura T. (1996). Prevalence of mood and Anxiety Disorders in a Working Population in Japan. JOEM.

[69] Eurofound and International Labor Organization (2019). Working Conditions in a Global Perspective.

[70] Toivanen S., Mellner C., Vinberg S. (2015). Selfemployed persons in Sweden—Mortality differentials by industrial sector and enterprise legal form: A five-year follow-up study. Am. J. Ind. Med..

[71] Lin S.-K., Lee C.-H., pan C.-H., Hu W.-H. (2003). Comparison of the prevalence of substance use and psychiatric disorders between government- and self-employed commercial drivers. Psychiatry Clin. Neurosci..

[72] Sônego M., Meller M., Massuti R., Campani F., Amaro J., Barbosa C., Rohde L.A. (2020). Exploring the association between attention-deficit/hyperactivity disorder and entrepreneurship. Rev. Bras. Psiquiatr..

[73] Peltonen J., Johansson E., Wincent J. (2020). Does attention-deficit hyperactivity disorder medication reduce entrepreneurship?. Health Econ..

[74] Verheul I., Rietdijk W., Block J., Franken I., Larsson H., Thurik R. (2016). The association between attention-deficit/hyperactivity (ADHD) symptoms and self-employment. Eur. J. Epidemiol..

[75] Anker E., Halmøy A., Heir T. (2019). Work participation in ADHD and associations with social characteristics, education, lifetime depression, and ADHD symptom severity. Atten. Deficit Hyperact. Disord..

[76] René Böheim U.M. (2009). Dependent Forms of Self-employment in the UK: Identifying Workers on the Border between Employment and Self-employment. J. Labour Mark. Res..

[77] Westerveld M. (2012). The ‘New’ Self-Employed: An Issue for Social Policy?. Eur. J. Soc. Secur..

[78] Horemans J., Marx I. (2017). Poverty and Material Deprivation among the Self-Employed in Europe: An Exploration of a Relatively Uncharted Landscape.

[79] Graeber D., Kritikos A.S., Seebauer J. (2021). COVID-19: A crisis of the female self-employed. J. Popul. Econ..

