# Supplementary material for: Occurrence of Mental Illness and Mental Health Risks among the Self-Employed: A Systematic Review
Source: Int J Environ Res Public Health. 2021 Aug 15;18(16):8617. doi: 10.3390/ijerph18168617 (PMC8393630; doi:10.3390/ijerph18168617)
Supplement: Supplementary file 1 [file ijerph-18-08617-s001.zip › ijerph-1280962-supplementary.pdf]

Supplementary Materials

Table S1. Quality assessment checklist for prevalence studies (adapted from Hoy et al) [1].

Name of author(s):
Year of publication:
Study title:

Risk of bias items	Risk of bias levels	Points scored	
1. Was the study`s target population a close representation of the national population in relation to relevant variables, e.g. age, sex, occupation?	**Yes (LOW RISK):** The study`s target population was a representation of the national population.	0	0
	**No (HIGH RISK):** The study`s target population was clearly NOT representative of the national population.	1	1
2. Was the sampling frame a true or close representation of the target population?	**Yes (LOW RISK):** The sampling frame was a true or close representation of the target population.	0	0
	**No (HIGH RISK):** The sampling frame was NOT a true or close representation of the target population.	1	1
3. Was some form of random selection used to select the sample, OR, was a census undertaken?	**Yes (LOW RISK):** A census was undertaken, OR, some form of random selection was used to select the sample (e.g. simple random sampling, stratified random sampling, cluster sampling, systematic sampling).	0	0
	**No (HIGH RISK):** A census was NOT undertaken, AND some form of random selection was NOT used to select the sample.	1	1
4. Was the likelihood of non-response bias minimal?	**Yes (LOW RISK):** The response rate for the study was ≥75%, OR, an analysis was performed that showed no significant difference in relevant demographic characteristics between responders and non- responders.	0	0
	**No (HIGH RISK):** The response rate was <75%, and if any analysis comparing responders and non-responders was done, it showed a significant difference in relevant demographic characteristics between responders and non-responders.	1	1
5. Were data collected directly from the subjects (as opposed to a proxy)?	**Yes (LOW RISK):** All data were collected directly from the subjects.	0	0
	**No (HIGH RISK):** In some instances, data were collected from a proxy.	1	1
6. Was an acceptable case definition used in the study?	**Yes (LOW RISK):** An acceptable case definition was used.	0	0
	**No (HIGH RISK):** An acceptable case definition was NOT used.	1	1
7. Was the study instrument that measured the parameter of interest (e.g. prevalence of low back pain) shown to have reliability and validity (if necessary)?	**Yes (LOW RISK):** The study instrument had been shown to have reliability and validity (if this was necessary), e.g. test-re- test, piloting, validation in a previous study, etc.	0	0
	**No (HIGH RISK):** The study instrument had NOT been shown to have reliability or validity (if this was necessary).	1	1
8. Was the same mode of data collection used for all subjects?	**Yes (LOW RISK):** The same mode of data collection was used for all subjects.	0	0
	**No (HIGH RISK):** The same mode of data collection was NOT used for all subjects.	1	1
9. Were the numerator(s) and denominator(s) for the parameter of interest appropriate?	**Yes (LOW RISK):** The paper presented appropriate numerator(s) AND denominator(s) for the parameter of interest (e.g. the prevalence of low back pain).	0	--
	**No (HIGH RISK):** The paper did present numerator(s) AND denominator(s) for the parameter of interest but one or more of these were inappropriate.	1	--
10. Summary on the overall risk of study bias	**LOW RISK**	0-3	0-2
	**MODERATE RISK**	4-6	3-5
	**HIGH RISK**	7-9	6-8

Table S2. Basic characteristics of **longitudinal and cross-sectional studies with moderate quality** on mental disorders comparing self-employed (s-empl) vs. employees (empl).

Author, publication date [reference]	Country/ region of study	Sample size, Female	Age [Mean (SD) or range]	Source population	Occupational groups	Disease outcomes: assessment tools
**Longitudinal study**						
**EUROPE**						
**Andersson 2008, (Recruitment: 1991, Follow-up: 10 y, Response: n.r.) [2]**	Sweden,	1,998, n.r.	s-empl: 41y empl: 37y	Swedish Level-of-Living Survey: representative random sample (1/1,000 of adult population)	s-empl vs empl	**Mental health problems:** (defined as sleeping problems, been tired, depressed, or anxious) questionnaire specially developed for the study
**Cross-sectional studies**						
**EUROPE**						
**Atherton 2007 [3]**	Great Britain	8,952, n.r.	45 y	Perinatal mortality register	s-empl (without personnel) vs s-empl (with personnel) vs empl (managerials/professionals)	**Anxiety and depression:** nurse-administered Clinical Interview Schedule
**Grégoris 2017 [4]**	France	437, 48% (s-empl) 42% (empl)	47(8.6) y (s-empl) 31.7(11.4) y (empl)	health-care insurance fund: self-employed and several occupational health services: employees convenience	s-empl (food service) vs empl (food service)	**1. Sleep disorders:** questionnaire specially developed for the study **2. Stress:** "do you feel stressed?" VAS 0-10
**Hounsome 2012 [5]**	Great Britain	784, 29.6 / 38.6% (farmers / non-farmers)	>16 y	Attendees of the Royal Welsh Agricultural Show and the Anglesey County Show in 2002 convenience	s-empl (non-farmers) vs s-empl (farmers) vs empl (non-farmers) vs empl (farmers)	**Psychological health:** 12-Item General Health Questionnaire (GHQ-12)
**Rugulies 2009 [6]**	Denmark	591, 58%	43(11) y	Danish Civil Registration System random	s-empl (low-grade) vs s-empl (high-grade) vs empl (non-manual high-grade)	**Depression, Anxiety, Somatization:** Hopkins Symptom Checklist 92 item version (SCL-92)
**ASIA**						
**Fujino 2005 [7]**	Japan	25,945, 40.5%	40-59 y	all living residents random	s-empl vs empl	**Occupational perceived stress:** 4-point scale

**Jamal 2007 [8]**	Canada (C), Pakistan (P)	554, 22% (C) 12% (P)	40 y (C) 38 y (P)	Participants of management programs in a local university, other potential participants convenience	s-empl vs empl (public, private organizations and part-time students)	**Burnout:** 22-item Maslach Burnout Inventory
**Kawakami 1996 [9]**	Japan	140, n.r.	>18 y	All persons living in Town A in Kofu-City random	s-empl vs empl (white-collar) vs empl (blue-collar)	**Any / depressive disorder:** Time-Ordered Stress and Health Interview (Diagnoses by DSM-III-R)
**Lewin-Epstein 1991 [10]**	Israel	276, 0%	25-65 y	Whole population of Holon (135.000 residents) and Bat-Yam (130.000 residents) random	s-empl vs empl	**Work-related stress:** questionnaire specially developed for the study
**Lin 2003 [11]**	Taiwan	1,011, 5.8% (s-empl) 3.2% (empl)	46.7(8.0) y (s-empl) 48.0(6.1) y (empl)	Routine biannual health check-up for renewal of commercial driver's license convenience	s-empl (drivers) vs empl (drivers)	**Non-psychotic neurotic symptoms:** Chinese Health Questionnaire (CHQ)
**Min 2019 [12]**	Korea	64,802, 35.2%	20-59 y	2008 Korean Community Health Survey (Korea centres for Disease Control and Prevention) register	s-empl (0-4 employees) vs s-empl (>5 employees) vs empl	**Suicidal ideation / Suicide attempts:** "yes/no"-question
**NORTH AMERICA**						
**Parasuraman 2001 [13]**	Pennsylvania	99 (s-empl) 287 (empl), 46.5% (s-empl) 51.2% (empl)	n.r.	adult students of evening Master of Business Administration courses convenience	s-empl vs empl (organizational)	**Life stress:** 10-item scale (Parasuraman et al. 1992)
**Prottas 2006 [14]**	New York	3,504, 56%	42.5(12.9) y	2002 National Study of the Changing Workforce (NSCW) random	s-empl (owners) vs s-empl (independants) vs empl	**Stress:** questionnaire specially developed for the study

SD = standard deviation, y = years, vs = versus, n.r. = not reported

Table S3. Results of cross-sectional studies on psychiatric disorders comparing self-employed (s-empl) vs employees (empl) of moderate or poor quality studies.

**Author, Publication date** [reference]	**Results**
	**Longitudinal study**
	**Self-rated poor general mental health**
Andersson 2008 [2]	**Mental health problems** s-empl vs empl:<br>1991: 20.1% vs 22.2%; 2000: 29.9% vs 33.0%<br>Mental health problems of empl (1991, no problems) who **move to s-empl** (2000) [FE (aSE)]: 0.493(0.340)*
	**Cross-sectional studies**
	**Any mental illness**
Grégoris 2017 [4]	**Sleep disorders**<br>s-empl vs empl: 37% vs 23.4% *
Kawakami 1996 [9]	**Any psychiatric disorders (e.g. panic, phobic)**<br>empl (white-collar) and empl (blue-collar) vs s-empl (ref.):<br>last six months: OR 2.23 (95%-CI 0.50-9.82) and 0.41 (95%-CI 0.03-4.51)<br>lifetime: OR 1.29 (95%-CI 0.44-3.74) and 0.65 (95%-CI 0.16-2.57)
	**Depression**
Rugulies 2010 [6]	**1. Depressive symptoms** s-empl (high-grade) and s-empl (low-grade) vs empl (high-level non-manual, ref.) [Mean(SD); Hopkins Symptom Checklist 92 item version, 13 items for depression (0 = no depression; 52 = extreme depression)]:<br>0.47 (0.44) and 0.58 (0.68) vs 0.45 (0.55); Beta (SE) 0.07 (0.17) and 0.14 (0.11)<br>**2. Severe depressive symptoms** s-empl vs empl (ref. = 5.2 %):<br>7.7% and 14.3%; OR 1.46 (95%-CI 0.16–13.73) and OR 2.97 (95%-CI 0.87–10.06)
Atherton 2007 [3]	**Depressive symptoms** s-empl vs empl (managerial/professional = ref.):<br>male: 6.0% vs 5.9%; OR 1.03 (95%-CI 0.70, 1.52), female: 8.7% vs 7.4%; OR 1.19 (95%-CI 0.76, 1.88)
Kawakami 1996 [9]	**Life time major depressive episode** s-empl (ref.) vs empl (white-collar) vs empl (blue-collar):<br>OR 1.16 (95%-CI 0.34-3.90) vs OR 0.66 (95%-CI 0.14-3.14)
	**Suicidal thoughts**
Min 2019 [12]	**1. Suicidal ideation**<br>s-empl (small business owner) vs s-empl (middle-large business owner) vs empl (ref.):<br>aOR 1.25 (95%-CI 1.15-1.35)* vs 1.32 (1.09-1.61)<br>**2. Suicidal attempt**<br>s-empl (small business owner) vs s-empl (middle-large business owner) vs empl (ref.):<br>aOR 1.67 (95%-CI 1.14-2.45)* vs 1.42 (0.51-3.98)
	**Anxiety / neurotic symptoms**
Rugulies 2009 [6]	**1. Anxiety symptoms** s-empl (high-grade) and s-empl (low-grade) vs empl (high-level non-manual, ref.) [Mean(SD); Hopkins Symptom Checklist 92 item version, 10 items for anxiety (0 = no anxiety; 40 = extreme anxiety)]:<br>0.27 (0.21) and 0.48 (0.58) vs 0.41 (0.49); Beta (SE) -0.12 (0.14) and 0.07 (0.09)<br>**2. Severe anxiety symptoms** s-empl (high-grade) and s-empl (low-grade) vs empl (ref. = 9.6%):<br>0% and 14.3%; -- and OR 1.58 (95%-CI 0.53–4.69)
Atherton 2007 [3]	**Anxiety** s-empl vs empl (professional/managerial = ref):<br>male: 4.6%; OR 0.84 (95%-CI 0.55, 1.29),5.4%, female: 7.0%; OR 0.98 (95%-CI 0.59, 1.60) vs 7.1

Lin<br>2003 [11]	**Non-psychotic neurotic symptoms** s-empl (drivers) vs empl (drivers) [Mean(SD); 12-item Chinese Health Questionnaire (cut-off point ≥ 4 = high risk group)]: 2.08(2.42) * vs 0.94(1.53)<br>CHQ ≥ 4: 7.5% vs 21.3% *
**Stress / Burnout / Exhaustion**	
Grégoris<br>2017 [4]	**Feeling stressed** s-empl vs empl: 57.5% vs 41.6% *<br>**Abnormal stress** s-empl vs empl: 17.8% vs 15.5%
Jamal<br>2007 [8]	s-empl vs empl (organizational) among Canadian (C) / Pakistani (P)<br>[Mean (F-value); 22-item Maslach Burnout Inventory (0 = no burnout; 132 = extreme burnout)]:<br>**1. Overall burnout** C: 38.13 vs 31.96 (18.84)* P: 33.23 vs 28.44 (8.99)*<br>**2. Emotional exhaustion** C: 26.13 vs 22.23 (15.59)* P: 23.11 vs 19.67 (12.86)*<br>**3. Depersonalisation** C: 9.97 vs 11.05 (1.89) P: 9.29 vs 8.87 (0.88)
Prottas<br>2006 [14]	**Stress** s-empl(without personnel) vs s-empl (with personnel) vs empl (ref.) [Mean(SD); 10-item 5 point Likert scale (10 = no stress; 50 = extreme Stress]:<br>0.04(0.6), 0.09 vs 0.03(0.6), Cohens d: 0.02 vs -0.01(0.56)
Fujino<br>2005 [7]	**Perceived stress** s-empl vs empl (data from 1988-90):<br>frequent: male: 11.3% vs 15.9%, female: 11.8% vs 15.1%<br>occasional: male: 11.0% vs 18.2%, female: 10.8% vs 15.1%
Parasuraman<br>2001 [13]	**Life stress** s-empl vs empl [Mean (SD); 10-item scale developed by the authors]: 2.87 (0.66) vs 2.82 (0.76)
Lewin-Epstein<br>1991 [10]	**Work-related stress** s-empl vs empl [Mean(SD); 7-point Likert scale (1 = no stress, 7 = extreme stress)]: 3.9 (2.3) vs 3.2 (2.1), effect (b)=0.04 *
**Self-rated poor general mental health**	
Hounsome<br>2012 [5]	**General health** s-empl vs empl [Mean(SE); 12-item General Health Questionnaire (0 = good general health; 36 = bad general health)]:<br>farmers: 10.66 (0.318) vs 10.46 (0.521)<br>non-farmers: 9.17 (0.518) vs 9.66 (0.255)

* $p<0.050$, vs = versus, ref. = reference, CI = confidence interval, OR = odds ratio, aOR = adjusted odds ratio, SD = standard deviation, SE = standard error, aSE adjusted standard error, FE = fixed effects

Table S4. **Overview of mental health outcomes** among self-employed compared to employed persons in longitudinal and cross-sectional observational studies with moderate or poor quality.

Study Design	Author, Publication date [reference]	Any mental illness	Depression or suicidal thoughts	Anxiety or Neuroses	Stress or Exhaustion or Burnout	Self-rated poor general mental health
Longi-tudinal	**EUROPE**					
	Andersson 2008 [2]					
Cross-sectional	**EUROPE**					
	Grégoris 2017 [4]					
	Hounsome 2012 [5]					
	Rugulies 2009 [6]					
	Andersson 2007 [2]				Exhaustion	
	Atherton 2007 [3]					
	**ASIA**					
	Min 2019 [12]		Suicidal thoughts			
	Jamal 2007 [8]				Burnout	
	Fujino 2005 [7]					
	Lin 2003 [11]			Neuroses		
	Kawakami 1996 [9]	**sole** / **non-sole**	**sole** / **non-sole**			
	Lewin-Epstein 1991 [10]					
	**NORTH AMERICA**					
	Parasuraman 2001 [13]					
	Prottas 2006 [14]					

The self-employed showed
= significantly lower occurrence
= lower occurrence
= no difference
= higher occurrence
= significantly higher occurrence
compared to the employed.

Self-employment subgroups:
"**sole**" = sole proprietorship (vs the employed)
"**non-sole**" = employing others (vs the employed)

References

1. Hoy, D.; Brooks, P.; Woolf, A.; Blyth, F.; March, L.; Bain, C.; Baker, P.; Smith, E.; Buchbinder, R. Assessing risk of bias in prevalence studies: modification of an existing tool and evidence of interrater agreement. *Journal of clinical epidemiology* **2012**, *65*, 934–939.

2. Andersson, P. Happiness and health: Well-being among the self-employed. *The Journal of Socio-Economics* **2008**, *37*, 213–236.

3. Atherton, K.; Power, C. Health inequalities with the National Statistics-Socioeconomic classification: disease risk factors and health in the 1958 British birth cohort. *European journal of public health* **2007**, *17*, 486–491.

4. Grégoris, M.; Deschamps, F.; Salles, J.; Sanchez, S. Health assessment of self-employed in the food service industry. *International journal of occupational and environmental health* **2017**, *23*, 234–242.

5. Hounsome, B.; Edwards, R.T.; Hounsome, N.; Edwards-Jones, G. Psychological morbidity of farmers and non-farming population: results from a UK survey. *Community mental health journal* **2012**, *48*, 503–510.

6. Rugulies, R.; Madsen, I.E.H.; Nielsen, M.B.D.; Olsen, L.R.; Mortensen, E.L.; Bech, P. Occupational position and its relation to mental distress in a random sample of Danish residents. *International archives of occupational and environmental health* **2010**, *83*, 625–629.

7. Fujino, Y.; Iso, H.; Tamakoshi, A.; Inaba, Y.; Koizumi, A.; Kubo, T.; Yoshimura, T. A Prospective Cohort Study of Employment Status and Mortality from Circulatory Disorders among Japanese Workers. *Journal of occupational health* **2005**, *47*, 510–517.

8. Jamal, M. Burnout and self-employment: a cross-cultural empirical study. *Stress and Health* **2007**, *23*, 249–256.

9. Kawakami, N.; Iwata, N.; Tanigawa, T.; Oga, H.; Araki, S.; Fujihara, S.; Kitamura, T. Prevalence of mood and Anxiety Disorders in a Working Population in Japan. *JOEM* **1996**, *38*, 899–905.

10. Lewin-Epstein, N.; Yuchtman-Yaar, E. Health Risks of Self-Employed. *Work and Occupations* **1991**, *8*, 291–312.

11. Lin, S.-K.; Lee, C.-H.; pan, C.-H.; Hu, W.-H. Comparison of the prevalence of substance use and psychiatric disorders between government- and self-employed commercial drivers. *Psychiatry and clinical neurosciences* **2003**, 425–431.

12. Min, J.-Y.; Kim, H.; Park, S.-G.; Hwang, S.H.; Min, K.-B. Differences in suicidal behaviors between self-employed and standardly employed workers. *American journal of industrial medicine* **2019**, *62*, 1144–1151.

13. Parasuraman, S.; Simmers, C.A. Type of employment, work-family conflict and well-being: a comparative study. *J. Organiz. Behav.* **2001**, *22*, 551–568.

14. Prottas, D.J.; Thompson, C.A. Stress, satisfaction, and the work-family interface: a comparison of self-employed business owners, independents, and organizational employees. *Journal of occupational health psychology* **2006**, *11*, 366–378.
